# Can Informal Care Be a Substitute for Formal Care? Evidence from Older People with Disabilities in Beijing, China

**DOI:** 10.3390/healthcare12242508

**Published:** 2024-12-11

**Authors:** Jiaoli Cai, Nan Jiang, Peter C. Coyte

**Affiliations:** 1 School of Economics and Management, Beijing Jiaotong University, No. 3 Shangyuancun, Haidian District, Beijing 100044, China; 2 Institute of Health Policy, Management and Evaluation, University of Toronto, Health Sciences Building, 155 College Street, Suite 425, Toronto, ON M5T 3M6, Canada; peter.coyte@utoronto.ca

**Keywords:** older people with disabilities, elder care, formal care, informal care, Beijing, long-term care, instrumental variables

## Abstract

Background: The demand for long-term care is increasing as the elderly population continues to grow, prompting a critical examination of care modalities. Methods: This study employs data from the Chinese Longitudinal Healthy Longevity Survey (CLHLS) to assess the relationship between informal and formal care for older people with disabilities in Beijing. The analysis employs the Probit model and incorporates the application of instrumental variable techniques and propensity score matching to ensure robustness in the results. Results: The findings highlight the important role of informal care. Conclusions: Policymakers should incorporate support policies for informal care into the overall design of the system, provide support to informal caregivers, and reduce their burden. Our research conclusions provide empirical reference for cities with similar characteristics to Beijing.

## 1. Introduction

Older people require specialized care due to their increased susceptibility to health issues when compared to younger demographics. This is particularly true for those with disabilities. Effective care systems are crucial for attaining high-quality population development, and long-term care typically involves both formal and informal aspects. Filial piety, often referred to as “孝” (xiào) in Chinese culture, is a fundamental virtue that emphasizes respect, obedience, and care for one’s parents and ancestors. As a cultural trait that traditionally emphasizes the family’s role in caring for older people, this has made informal practices a key component of Chinese elder care [1,2]. As the demand for elder care in China continues to grow, it is essential to further clarify the relationship between formal and informal care systems.

Beijing is one of the most representative cities in China with an aging population trend. In 2023, Beijing’s permanent population aged 60 and above was 4.948 million, an increase of 6.4% from 2022; this accounts for 22.6% of the city’s total population and is 1.5 percentage points higher than the national average [3]. In addition, Beijing’s old-age dependency ratio, which measures the number of older people (typically age 65 and older) as a proportion of the working-age population (usually ages 15–64), is continuing to rise. From the perspective of permanent population, according to the population aged 15–59 supporting older people aged 60 and above, the old-age dependency ratio is 34.6%, which means that it takes 2.9 people in the permanent labor force to support one older person [3]. The substantial number of older people with disabilities creates a high demand for care.

The relationship between formal and informal care is more complex in Beijing than in other cities. On one hand, the city boasts abundant medical resources, numerous health care institutions such as nursing homes, and a well-developed network of social services. Families in higher socio-economic groups tend to lean toward formal care options. On the other hand, Beijing’s rich cultural heritage and strong moral traditions make informal care an equally significant option. This unique combination of factors makes Beijing an ideal subject for studying the intricate dynamics between informal and formal care in metropolitan areas.

Formal care refers to organized, market-based, and paid services provided by professional personnel in institutions such as hospitals and nursing homes. Informal care refers to unorganized, non-market, and unpaid services provided by non-professional caregivers, typically family members or friends [4]. Research on the relationship between formal and informal care generally falls into two perspectives: substitution and complementarity. The substitution view suggests that informal care, influenced by factors such as family dynamics and the caregiver’s participation in the labor market, acts as the alternative to formal care [5]. Zhang et al. (2020) conducted a study in Shanghai which indicated that a significant amount of informal care is replaced by formal care [6]. Zang (2022) analyzed data from the CLHLS 2018 to find that informal care can act as a substitute for formal care [2]. Perdrix and Roquebert (2022) found that an increase in the amount of formal care is associated with a slight decrease in the likelihood of using informal care [7]. Lyu et al. (2024) compared the relationship between informal and formal social care among older people in England before and after the implementation of the Care Act 2014, and the results suggest that receiving informal care can be a substitute for receiving formal care [8]. Conversely, the complementarity perspective posits that both informal and formal practices increase simultaneously in response to rising demand for long-term care [9,10]. Liu (2021) analyzed data from the China Nutrition and Health Survey (CNHS) from 1989 to 2015, finding that the complementary effect of formal care on informal care outweighs the substitution effect [11]. Rapp et al. (2022) investigated the impact of formal care expenditure on the use of informal care among older adults in Europe, and the conclusion is that formal and informal care are complementary in the early stages of the dependency process [12].

In countries like France and Japan, the right to receive formal care is independent of whether informal care is provided; receiving informal care does not affect eligibility for government support. In contrast, in places like the UK and Australia, the amount of informal care provided by family members is factored into assessments of eligibility for government support [13].

Mathematical models have become well-established as research methods, providing a strong foundation for understanding the relationship between formal and informal care [14,15]. However, the precise functional forms of these models have not been determined, making it difficult to theoretically prove the relationship [4,14]. Empirical testing, such as causal inference methods, is still needed to clarify whether formal and informal care are substitutes or complements, as this remains essentially an empirical issue [4].

In econometric analyses, there is a general agreement that endogeneity issues arise due to the mutual causality between formal and informal care. Most studies have addressed this using instrumental variable methods, with various instruments like the number of children, number of daughters, and the geographical proximity of children to their parents, among others [4]. Additionally, the boundary between formal and informal care may be unclear. For example, there is debate regarding how to classify “social services”. We define social services as elder care provided by the government, communities, and nursing institutions, categorizing them as a form of formal care.

Variability in the relationship between informal and formal care is likely influenced by differences in regions, time periods, data sources, and methodologies across studies, as well as by the specific purposes for which care is utilized. In general, medical services are used to restore or maintain health, while long-term care serves to improve well-being by supporting daily activities [16]. When care recipients require high-quality, professional medical services, informal care may not effectively substitute for formal care. However, when the focus is on routine caregiving tasks to assist with daily activities, the relationship between informal and formal care becomes ambiguous [4]. Further, most existing studies adopt a national perspective; few studies examine region-specific dynamics. Studies also tend to rely on cross-sectional data, which limits the ability to establish causal relationships. It is of great significance to focus on the care of older people with disabilities in specific areas, such as large cities like Beijing. As the capital of China, Beijing has a special leading position in policy formulation and practical exploration. Its governance practices in dealing with the risks of elderly disability care can provide valuable experience for other large cities. The use of longitudinal data can help to further identify causal relationships.

Therefore, this study focused on older people with disabilities in Beijing and used longitudinal data to explore the impact of informal care on formal care. Data were from five waves of the Chinese Longitudinal Healthy Longevity Survey (CLHLS) between 2005 and 2018. We addressed endogeneity concerns by employing instrumental variable and propensity score matching methods. Our goal is to narrow gaps in previous studies that have overlooked regional variations, securing valuable insights for shaping long-term elder care policies.

## 2. Materials and Methods

### 2.1. Data Sources and Sample Selection

Data from the CLHLSs conducted in 2005, 2008, 2011, 2014, and 2018 were utilized in this study. The baseline survey of the CLHLS was conducted in 1998, with a total of eight surveys carried out from 1998 to 2018 (specifically in 1998, 2000, 2002, 2005, 2008, 2011, 2014, and 2018). The survey covers 23 provinces, municipalities, and autonomous regions in China. These include Heilongjiang, Jilin, Liaoning, Beijing, Tianjin, Hebei, Henan, Shanxi, Shandong, Shaanxi, Jiangsu, Zhejiang, Shanghai, Fujian, Anhui, Jiangxi, Hunan, Hubei, Sichuan, Chongqing, Guangdong, Guangxi, and Hainan, encompassing approximately 85% of the national population and providing a sound representation. The response rate for each survey is about 90%, and sample loss is relatively low [17,18].

China issued the Opinions on Accelerating the Development of the Elderly Care Service Industry in 2006, marking the nation’s first policy to introduce the concept of an elder care service system. From 2005 onwards, the CLHLS included questions regarding care provided by children, grandchildren, and spouses. Therefore, we selected 2005 as the starting point for this research.

While the CLHLS includes questions about the provision of care to older people with impairments in one or more of six daily activities—bathing, dressing, using the toilet, indoor mobility, controlling bowel and bladder functions, and eating—our focus is on those who are disabled. As a consequence, older people (65 years and older) with impairments in one or more of these six activities were classified as disabled for the purposes of this analysis. After applying this inclusion criteria, 876 older people with disabilities in Beijing were identified. Furthermore, we deleted the missing values of key variables, such as formal care variables and informal care time variables, and finally kept 590 samples.

### 2.2. Variables

#### 2.2.1. Dependent Variable

The dependent variable in this study was the use of formal care by older people with disabilities in Beijing, measured through two questions. The first question is, “Who are you currently living with?” If the response is “living in a nursing home”, this indicates the use of nursing home services and therefore, formal care. Responses such as “living with family” or “living alone” indicate that nursing home services are not being used and thus that formal care is not being received. The second question is, “When you need help with daily activities (E1, E2, E3, E4, E5, and E6), who is your main helper?” If the response is “a caregiver or social service”, this indicates the use of formalized domestic services, while responses such as “spouse”, “other relatives”, or “neighbors” indicate that formal domestic care is not being received. If either nursing home services or domestic formal services are used, the value is assigned as 1; otherwise, it is 0.

#### 2.2.2. Independent Variable

The central independent variable for this study is the time spent on informal caregiving services, derived from the questionnaire item: “In the past week, how many total hours have your children/grandchildren and other relatives provided you with daily care assistance?”.

#### 2.2.3. Control Variables

To eliminate the estimation bias caused by omitted variables, control variables were selected based on the Anderson Health Behavior Model. These include predisposing factors such as age, gender, and education; enabling factors like place of residence, health insurance coverage, and whether an individual lives with a spouse; and needs-based factors, which include the degree of disability measured by activities of daily living (ADL), counting impairments in the six activities listed above (e.g., bathing, eating). Social disability is measured using instrumental activities of daily living (IADL), which counts impairments in eight activities: shopping, cooking, doing laundry, visiting neighbors, walking continuously for 1 km, lifting 5 kg, squatting and standing three times, and using public transportation. A higher number of ADL impairments indicates a higher degree of physical disability; similarly, a higher number of IADL impairments indicates a higher degree of social disability.

#### 2.2.4. Instrumental Variable

As mentioned above, there is an endogeneity issue created by the mutual causality between informal care and formal care usage. We employed the instrumental variable method to correct for endogeneity. With reference to the literature, we selected the distance of the nearest child’s residence as the instrumental variable [4,14,19]. The distance between children’s residence and their parents’ place of residence would affect the time cost of their children providing care, thereby affecting their children’s decision to provide informal care. At the same time, it would not directly affect the amount of formal care used by parents, making it an effective instrumental variable. According to the CLHLS, the distance of the nearest child’s residence is categorized into five levels: living together, in the same village, in the same town or district, in the same county or city, or in the same province. For our analysis, if the distance is living together, it was coded as 0; if in the same village, as 1; if in the same town or district, as 2; if in the same county or city, as 3; and if in the same province, as 4. A larger value indicates a greater distance.

The specific definitions and measurements of all variables are listed in Table 1.

### 2.3. Model Design

We constructed the decision equation for the utilization of formal care services for older people with disabilities based on the Anderson Health Behavior Model as follows:

(1) $${\mathrm{Formal}}_{\mathrm{it}}=\alpha_{0}+\alpha_{1}{\mathrm{Informal}}_{\mathrm{it}}+\alpha_{2}X_{\mathrm{it}}+\mu_{\mathrm{it}}$$

where the dependent variable Formal indicates whether older people with disabilities had adopted formal care, represented as a binary variable. The independent variable Informal measures the time spent on informal care services in hours. X represents a series of predisposing, enabling, and needs-based factors, and *μ* is the error term.

## 3. Results

### 3.1. Descriptive Statistics

Table 2 reports the descriptive statistics of the sample. On average, informal care time was 52.07 h per week, indicating a high level of care intensity. Most individuals had children living nearby, providing a solid foundation for informal care. Regarding predisposing factors, the sample was relatively older, with an average age of about 88 years; males account for about 41% of the sample and about 64% had received some level of education. In terms of enabling factors, 73.75% lived in the city, 32.27% were married and cohabiting with their spouses, and about 65% had health insurance coverage. In terms of needs-based factors, the average number of ADL impairments was more than 4, and the average number of IADL impairments was about 3, indicating a high overall level of disability.

### 3.2. Analysis of Formal and Informal Care Usage

**Figure 1:**
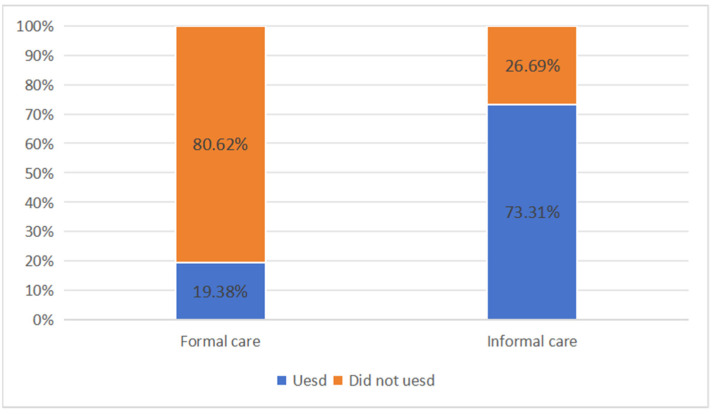
Distribution of formal care and informal care usage. Note: If the informal care time was greater than 0, it means that informal care was used. If the informal care time was 0, it means that informal care was not used.

Figure 1 shows the statistics for formal and informal usage in the sample. The proportion of older people with disabilities using formal care was less than 20%, while those using informal care services reached 73.31%. Overall, long-term care for older people with disabilities in Beijing predominantly relies on informal systems.

### 3.3. Regression Results

Table 3 reports the results of the baseline regression and the instrumental variable regression for the determinants of the use of formal care. Column 1 reports the Probit model where informal care time is treated as an exogenous variable. This regression result demonstrates that informal care was significantly negatively correlated with the utilization of formal care.

To eliminate potential endogeneity, we used the nearest living distance of children as an instrumental variable (IV) and estimated the determinants of the use of formal care with an IV-Probit model. The Wald test value for endogeneity is 49.46 (*p <* 0.0001), which rejects the null hypothesis “the model does not have endogeneity” at the 1% significance level, confirming the validity of the IV-Probit model.

The IV-Probit model results (see column 2) for the determinants of the use of formal care demonstrate that an increase in informal care significantly reduced the likelihood of using formal care for older people with disabilities. Additionally, the results from other control variables show that older people with higher levels of physical and social disability were less likely to use formal care; those living with a spouse also had a lower likelihood of using formal care systems. Men had a reduced likelihood of using formal care compared to women.

### 3.4. Heterogeneity Test

A further analysis of the impact of informal care on the use of formal care services, under constraints such as gender, degree of disability, and residence, is presented in Table 4. The degree of disability was categorized as 1 to 2 ADL impairments indicating mild disability, 3 to 4 indicating moderate disability, and 5 to 6 indicating severe disability. The results show that, for both men and women in the sample, informal care services significantly substitute for formal care services. However, formal care needs vary substantially based on the degree of disability. For those with severe disabilities, specialized formal care is a rigid necessity and informal care is unsuitable. Concerning urban–rural differences, urban residents exhibit a more pronounced substitution effect of informal care for formal care.

### 3.5. Robustness Test

#### 3.5.1. Replacing Explanatory Variable

To ensure the robustness of our findings, we repeated the analysis but replaced the key independent variable, informal care time (which is a measure of intensity), with “whether informal care is used”, which captures the extensive margin and is a binary variable. The use of informal care was based on the responses to “Who primarily takes care of you when you are unwell or sick?” and “If you encounter problems or difficulties, who do you think of first to help you solve them?” If the respondent answered at least one of those two questions with a spouse, (grand)children and their spouses, other relatives, or friends and neighbors, the binary variable was assigned a value of 1; otherwise, it was assigned a value of 0. The regression results, as shown in Table 5, continued to exhibit a significant negative correlation, confirming the reliability of the previous findings.

#### 3.5.2. Propensity Score Matching

We initially used the instrumental variable method to address potential endogeneity issues. However, this approach is controversial and selecting appropriate instrumental variables can be challenging. Therefore, we further employed the propensity score matching (PSM) method to address the endogeneity between informal and formal care.

Specifically, the treatment group and control group were distinguished based on the time spent on informal care, with the treatment group defined as those with an informal care time greater than zero and the control group as those with an informal care time equal to zero. Next, individual socio-economic characteristics (age, gender, education, place of residence, health insurance coverage, and cohabitation with a spouse) were used to construct a “counterfactual scenario”. Finally, the average treatment effect of informal care on formal care was calculated. Because the sample data were primarily based on observations from 2018, only observations for 2018 were used for PSM to avoid errors.

Table 6 reports the average treatment effects under different matching methods; these results have passed a balance test. Regardless of the matching method used, informal care exhibits a significant suppressive effect on formal care. This is consistent with the results drawn from the instrumental variable method and further confirms the substitutive role of informal care for formal care.

## 4. Discussion

This study, based on data from older people with disabilities in Beijing, found that informal care has a significant substitution effect on formal care. These findings align with those of Zhang et al. (2020) based on data from Shanghai, suggesting that the present study’s results may be applicable to other large cities as well [6]. Our findings are also consistent with findings of studies from developed countries in Europe and the United States [2,6,7,8]. However, the results diverge from those of Liu (2021), who used data from the China Health and Nutrition Survey from 1998 to 2005 to employ a two-stage stochastic frontier model, finding that the complementary effect of formal care on informal care outweighs its substitution effect [11]. The difference in results is likely influenced by differences in data and modeling choices, as well as the geographic scope of the study area.

While our findings are consistent with previous studies, it is important to consider the type of formal care in the analysis. This study defined care provided by nursing homes, caregivers, and social services as formal care, and care provided within the family as informal care. Although these definitions are relatively narrow compared to research in Western countries, these forms of care are the most important and common in China. From a global perspective, these forms of care hold a central position within the realm of long-term care, rendering our research conclusions a valuable reference for addressing the global challenge of caring for an older population with disabilities. In addition, the ways of caregiving are becoming increasingly diversified. Scholars have discussed the concepts of family solidarity, the de-familialization of care, and the de-gendering of care, which may further blur the distinction between formal and informal caregiving [20].

The endogeneity of informal care could vary depending on the type of formal care. For example, Bolin et al. (2009) rejected the exogeneity hypothesis of informal care only in the case of formal family care, but did not find sufficient evidence to reject it for doctor visits and overnight hospitalization [4]. Conversely, Van Houtven and Norton (2004) found that informal care is endogenous in determining formal home care, nursing home care, physician visits, and hospital care [14].

Informal care generally incurs lower pension costs, so encouraging its use could reduce overall pension expenditures and lead to a more efficient allocation of resources for elder care [21]. Therefore, the development of informal care systems represents an effective response to older people. The government should provide more supportive policies for informal care. Specifically, the government could offer cash subsidies to family members for caregiving to alleviate the financial burden and provide skills training to enhance the caregiving abilities of family members. However, this does not imply that policymakers should focus solely on informal care. The growing older population and constraints on public spending for older people with long-term health issues in China may lead to an over-reliance on informal care as a solution for long-term care [22].

The use of formal and informal care in China is in a state of imbalance. China has always emphasized the establishment of a comprehensive long-term care system, supporting the construction of facilities such as nursing homes and community day care centers for older people with disabilities, and establishing subsidy systems for economically disadvantaged and disabled older people. China gradually established a long-term care insurance system after 2016, primarily covering the costs of formal care services provided by communities and institutions. However, based on our study sample, the utilization of formal care is not high. Although socio-economic development and shifting family structures have diminished the traditional role of family support, social services for older people have not completely replaced this function. As a result, informal care continues to be the dominant form of long-term care, with older people with disabilities struggling to access formal care services [2,13,23].

It is also important to note that formal and informal care serve different functions. As per the heterogeneity analysis, for severely older people with disabilities (who account for up to 70% of the sample), informal care does not readily substitute for formal care. Formal care in these cases is better equipped to handle the heavier burdens and high technological requirements associated with severe impairments, and informal care practices alone cannot meet the needs of this population. Similarly, Bonsang (2009) found that the substitutability of informal care diminishes as disability levels increase [21]. These findings imply that the role of informal care in the long-term care system is limited when addressing the needs of severely older people with disabilities.

Our analysis of rural–urban heterogeneity also revealed that informal care in urban areas has a more significant substitution effect than in rural areas, as expected. In urban settings, the cost of formal care is likely higher compared to the opportunity cost of informal care provided by family members, leading to a greater reliance on informal care. In contrast, rural areas face a shortage of caregivers due to the migration to many urban centers; the physical distance between older people and their caregivers is often much greater in these areas. These factors create a stronger demand for formal care in rural areas [24]. Previous studies had also shown that rural residents tend to face more unmet care needs and have less access to care services than their urban counterparts [25,26].

Based on the above discussion, we think that the substitution relationship between informal and formal care does not imply that these forms of care are separate entities. The government should play a leading role in clearly defining the functions of both, ensuring appropriate divisions of labor and making policy adjustments to address the needs of different populations and regions. For example, long-term care insurance should provide support for caregiving within families; the development of formal care services should focus on rural older people with disabilities and older people with severe disabilities.

This study has several limitations. First, although we compared our study with other relevant research, certain inherent characteristics of Beijing as a specific region, such as being the political and economic center of China, may limit the generalizability of our conclusions. Second, due to the large number of missing values for potential factors such as economic factors, we did not use them as control variables. Excluding them may lead to an overestimation of the effects of informal care on formal care. Last, this study did not differentiate between the various types of formal and informal care. As mental health among older people is receiving increasing attention, future studies could explore the division of long-term care into both physiological and psychological practices.

## 5. Conclusions

As the population rapidly ages, the construction and improvement of long-term care systems is becoming increasingly crucial. This study used data from five waves of the CLHLS (2005, 2008, 2011, 2014, and 2018), employed the instrumental variables method to address endogeneity, and applied PSM to ensure the robustness of the results. The findings suggest that informal care has a significant substitution effect on formal care for older people with disabilities in Beijing. Policymakers should focus on the establishment of an informal care system to reduce the social costs of aging and strike a balance between informal and formal care practices, particularly for severely older people with disabilities and those living in rural areas. In addition, it is important to explore the new policies for elder care alongside innovations in related technologies and concepts.

## Figures and Tables

**Table 1:** Description of variables.

Variable Category	Variable Classification	Variable Name	Variable Settings
Dependent variable		Formal care	Using home care services or nursing home services = 1, No = 0
Independent variable		Informal care time	According to the response to the question in the CLHLS questionnaire, “In the past week, how many total hours have your children/grandchildren and other relatives provided you with daily care assistance?”
Instrumental variable		The nearest living distance of children	The same residence = 0, same village = 1, same town or district = 2, same county or city = 3, same province = 4
Control variables	Predeposing factors	Age	Age of respondents
		Gender	Male = 1, Female = 0
		Education	Attended school = 1, Did not attend school = 0
	Enabling factors	Marriage	Married and living with spouse = 1, otherwise = 0.
		Place of residence	Urban = 1, Rural = 0.
		Health insurance	Have = 1, Do not have = 0
	Needs-based factors	Degree of disability	The number of impaired activities of daily living
		Degree of social disability	The number of impairments in instrumental activities of daily living

**Table 2:** Descriptive statistics.

Variable	Obs.	Mean/ Percentage	Std.	Min	Max
Formal care	872		0.396	0	1
0		80.71			
1		19.29			
Informal care time	738	52.07	70.96	0	641
Nearest living distance of children	733		1.204	0	4
0		38.94			
1		21.17			
2		18.72			
3		19.67			
4		1.49			
Age	876	88.06	11.31	65	111
Gender	876		0.492	0	1
0		58.86			
1		41.14			
Education	876		0.481	0	1
0		36.14			
1		63.86			
Place of residence	876		0.440	0	1
0		26.25			
1		73.75			
Health insurance	840		0.476	0	1
0		34.60			
1		65.40			
Marriage	870		0.467	0	1
0		67.73			
1		32.27			
Degree of disability	876	4.696	1.569	1	6
Degree of social disability	876	2.947	3.278	0	8

Note: The unit of this study sample is the individual. These characteristics are the summary statistics across waves. Mean is presented for continuous variables, and percentage is presented for categorical variables. For formal care, using home care services or nursing home services = 1, no = 0. For the nearest living distance of children, the same residence = 0, same village = 1, same town or district = 2, same county or city = 3, same province = 4. For gender, male = 1, female = 0. For education, attended school = 1, did not attend school = 0. For place of residence, urban = 1, rural = 0. For health insurance, have = 1, did not have = 0. For marriage, married and living with spouse = 1, otherwise = 0.

**Table 3:** Results of regression.

	Probit Model	IV-Probit Model
Informal care time	−0.004 *** (−2.72)	−0.055 *** (−3.79)
Degree of disability	−0.096 ** (−2.04)	−0.581 *** (−3.38)
Degree of social disability	−0.097 *** (−3.18)	−0.253 *** (−3.14)
Age	−0.014 (−1.60)	0.047 * (1.68)
Gender: Male (reference group: Female)	−0.067 (−0.48)	−0.778 ** (−2.08)
Married and living with a spouse (reference group: others)	−0.783 *** (−4.17)	−0.896 ** (−2.32)
Education: Attended school (reference group: Did not attend school)	0.055 (0.34)	0.0186 (0.05)
Health insurance: Yes (reference group: No)	−0.097 (−0.74)	−0.164 (−0.52)
Residence: Urban (reference group: Rural)	0.770 *** (4.67)	0.0248 (0.06)
Year: 2008	0.052 (0.21)	NA
Year: 2011	0.254 (0.94)	−1.254 * (−1.70)
Year: 2014	0.501 (1.55)	−0.0112 (−0.01)
Year: 2018	0.615 *** (3.10)	0.0238 (0.06)
Constant term	0.408 (0.46)	1.614 (0.72)
Observation	703	590
Wald test		49.46 ***

Note: (1) *, **, *** denotes significance at the 10%, 5%, and 1% levels, respectively. (2) For the Probit model, the values in parentheses were t-values. For the IV-Probit model, the values in parentheses were z-values. NA means that the regression results of 2008 were directly omitted in the regression process. This is because the sample size of 2008 in the year variable was too small.

**Table 4:** Heterogeneity regression result.

	Gender		Degree of Disability			Residence	
	Female	Male	Mild	Moderate	Severe	Rural	Urban
Informal care time	−0.003 * (−1.87)	−0.007 *** (−2.67)	−0.007 *** (−3.02)	−0.008 *** (−3.34)	−0.001 (−0.81)	−0.004 (−1.20)	−0.004 ** (−2.48)
Control variables	Yes	Yes	Yes	Yes	Yes	Yes	Yes
Constant	0.1195 (0.10)	0.719 (0.50)	−3.797 (−1.43)	−1.295 (−0.60)	1.150 (1.01)	5.955 ** (2.33)	0.851 (0.89)
Observation	421	282	116	100	487	140	524

Note: Control variables were the same as in Table 3 and their results were omitted; *, **, *** indicate significance at the 10%, 5%, and 1% levels, respectively; z-values are in parentheses.

**Table 5:** Results for replacing independent variable.

	Probit Model
Whether to use informal care: Yes (Reference group: No)	−1.790 *** (−10.37)
Control variables	Yes
Constant	0.540 (0.43)
Observation	590

Note: Control variables were the same as in Table 3 and their results were omitted; *** indicate significance at the 1% level; z-values are in parentheses.

**Table 6:** Average treatment effect.

Matching Method	Treatment Group	Control Group	Difference	Standard Error	*t*-Test Value
Nearest neighbor matching	0.18	0.376	−0.196	0.0543	−3.60
Caliper matching	0.18	0.376	−0.196	0.052	−3.80
Kernel matching	0. 18	0.382	−0.202	0.052	−3.88

Note: Neighborhood matching was carried out using a pair of four matches; caliper matches had a radius of 0.05; kernel matches had a bandwidth of 0.05.

## Data Availability

Data are contained within the article. The datasets used and analyzed during the current study are available from Peking University Open Research Data Platform: https://opendata.pku.edu.cn/ (accessed on 15 October 2024).
